# Efficacy of glyburide vs. metformin in preventing neonatal birth obesity in pregnancies complicated by gestational diabetes mellitus: a systematic review and meta-analysis

**DOI:** 10.3389/fped.2025.1604572

**Published:** 2025-09-26

**Authors:** Wan Jiang, Hongli Zhang, Xin Liu, Jianglan Xie, Jian Liu

**Affiliations:** ^1^ Department of Pharmacy, Chengdu Shuangliu District Maternal and Child Health Hospital, Chengdu, Sichuan, China; ^2^ Department of Women Health Care, Chengdu Shuangliu District Maternal and Child Health Hospital, Chengdu, Sichuan, China

**Keywords:** gestational diabetes mellitus, glyburide, metformin, macrosomia, birth weight

## Abstract

**Background:**

Gestational diabetes mellitus (GDM) has significant implications for both maternal and fetal health, increasing the risk of macrosomia, neonatal hypoglycemia, and long-term metabolic complications in offspring. Given these concerns, a comprehensive evaluation of treatment options, including glyburide and metformin, compared to insulin, is essential to guide clinical practice.

**Methods:**

A systematic literature search was conducted following the Preferred Reporting Items for Systematic Reviews and Meta-Analyses (PRISMA) guidelines, utilizing PubMed, EMBASE, and Web of Science without restrictions on date or language. The focus was on studies comparing oral medications (glyburide or metformin) with insulin for GDM, assessing outcomes, e.g., birth weight and the risk of macrosomia. Studies with non-relevant study designs were excluded. Data extraction and management were conducted with bias assessment using SYRCLE's tool. Statistical analyses were performed using R, incorporating both fixed and random effects models, subgroup analyses, and tests for publication bias.

**Results:**

This meta-analysis reviewed 23 studies (20 randomized controlled trials and 3 retrospective cohort studies) that evaluated treatments for GDM. The overall risk of macrosomia did not differ statistically between oral medications and insulin [odds ratio (OR) = 0.8534, 95% confidence interval (CI) (0.6271; 1.1614), *p* = 0.3134]. However, subgroup analysis revealed that glyburide increased the risk (OR=1.3806, *p* < 0.05), whereas metformin reduced it (OR = 0.6728, *p* < 0.0001). No statistical difference was found in infant birth weights between oral medications and insulin [mean difference (MD) = 14.3838, 95% CI (−40.7746; 69.5421), *p* = 0.6093], but subgroup analysis indicating that glyburide increased birth weight [MD = −83.32, 95% CI (−160.74 to −5.91)], and metformin decreased it [MD = 72.80, 95% CI (26.24–119.36)].

**Conclusion:**

This meta-analysis suggests that oral medications for GDM do not statistically alter the overall risk of macrosomia or infant birth weight compared with insulin administration. However, glyburide is associated with an increased risk of macrosomia, whereas metformin appears to reduce this risk. Consistent with these findings, glyburide was associated with an increase in infant birth weight, while metformin was associated with a decrease. These findings emphasize the importance of personalized treatment strategies for GDM management.

## Introduction

Gestational diabetes mellitus (GDM) presents a large health challenge, impacting not only the immediate well-being of pregnant women but also posing long-term risks to their offspring. This condition increases the likelihood of fetal macrosomia, which is associated with higher rates of birth injuries, asphyxia, neonatal hypoglycemia, and hyperinsulinemia (1). Uncontrolled GDM further exacerbates these risks, leading to accelerated fetal growth, excessive fat accumulation, insulin resistance, and an increased predisposition to future obesity and type 2 diabetes in the offspring (2). Given these concerns, understanding the impact of therapeutic interventions is crucial for mitigating these health risks.

In response to the challenges posed by GDM, newer oral hypoglycemic agents, e.g., metformin and glyburide, have emerged as alternatives to insulin therapy. These medications have demonstrated efficacy in managing blood glucose levels during pregnancy. However, concerns remain regarding their long-term safety for both mothers and their children. While short-term childhood outcomes appear comparable between these oral agents and insulin, emerging evidence suggests that in-utero exposure to these drugs may have lasting metabolic effects and increase susceptibility to various health conditions later in life (3). This uncertainty highlights the need for comprehensive research to better understand the balance between the benefits and potential risks of these treatment options.

The association of metformin with a reduced incidence of neonatal hypoglycemia is promising; however, it has also been linked to potentially increased risks of low birth weight and preterm birth—outcomes that remain debated and may vary depending on the specific clinical context in which metformin is used (4). These complexities underscore the challenges of effectively managing GDM. Consequently, conducting a meta-analysis to synthesize existing research is essential. Such an analysis can provide a clearer understanding of how these medications compare with insulin in terms of efficacy, immediate neonatal outcomes, and potential long-term effects on both the child and mother. Thus, we performed this meta-analysis to fill in these knowledge gaps, ultimately informing clinical practice with evidence-based recommendations for GDM management.

## Methods

### Search strategy

We conducted a systematic literature search following the guidelines of the PRISMA statement (5). Our search included databases such as EMBASE, PubMed, and Web of Science, using a comprehensive set of keywords and Medical Subject Headings (MeSH) terms related to GDM, oral medications (specifically, glyburide and metformin), insulin therapy, infant birth weight, and macrosomia. The search strategy combined terms such as “*gestational diabetes,”* “*oral hypoglycemic agents,”* “*glyburide,”* “*metformin,”* “*insulin,”* “*birth weight,”* and “*macrosomia”* using Boolean operators.

To ensure a broad and inclusive selection of relevant literature, we applied no language or date restrictions in our search. Additionally, we manually reviewed the reference lists of key articles and systematic reviews to identify any other potentially relevant studies.

### Inclusion and exclusion criteria

Studies were included if they met the following criteria: participants were pregnant women diagnosed with GDM; interventions involved a comparison of oral medications (glyburide or metformin) with insulin; outcomes included infant birth weight or macrosomia risk; and the study design was either a randomized controlled trial (RCT) or a retrospective cohort study. Studies were excluded if they did not involve human participants, were unrelated to GDM, were reviews, meta-analyses, or case reports, or lacked sufficient data for analysis. Additionally, studies with unsuitable designs, such as case series or cross-sectional studies, or those in which data could not be extracted for meta-analysis due to format or completeness issues, were also excluded.

### Data collection and management

Data extraction was conducted independently by two reviewers using a standardized form to collect information on study characteristics (author, year, and study design), participant demographics, intervention details (type of oral medication vs. insulin), and outcomes (MD in birth weight and OR for macrosomia). Discuss until an agreement was reached, or consult a third reviewer, when there were discrepancies. All data were managed using a secure database following the guidelines provided by Balduzzi et al., 2019 (6). The study data were cross-checked with the original articles to ensure accuracy, and when necessary, the authors were contacted for clarification or to obtain additional data.

### Bias assessment methodology in included studies

Bias in studies on treatments for gestational diabetes was assessed using SYRCLE's risk of bias tool, which evaluates eight key domains: blinding of outcome assessment, allocation concealment, random sequence generation, incomplete outcome data, blinding of participants and personnel, selective reporting, other sources of bias, and overall assessment. Random sequence generation and allocation concealment were examined to ensure unbiased selection of treatment groups. Blinding of participants, personnel, and outcome assessors was performed to mitigate performance and detection biases. Incomplete outcome data were evaluated to prevent attrition bias, and selective reporting was scrutinized to avoid biases in reported outcomes. Additional potential biases were considered under the category of “other sources of bias.”

“low,” “high,” or “unclear” risk of bias was defined for each domain. To aid in visualizing these assessments, we utilized the Risk-of-bias VISualization (robvis) tool, an R package, and Shiny web application described by McGuinness and Higgins in their 2021 publication (7). This tool provides graphical representations that enhance the interpretation of bias risk across the studies.

### Statistical analysis

R (Version 4.3.1) was used for statistical analysis. Both random- and fixed-effects models were employed. For continuous outcomes, such as infant birth weight, we calculated both 95% CI and MD. The MD was calculated as the control group mean minus the experimental group mean. Therefore, a negative value indicates an increase in the outcome measure for the experimental group, while a positive value indicates a decrease. For dichotomous outcomes, such as macrosomia risk, the Mantel-Haenszel method was used for determination of ORs. Heterogeneity was assessed using the I^2^ statistic, tau^2^ statistic, and Q-statistic. Subgroup analyses were performed to compare glyburide and metformin separately with insulin. To assess publication bias, we implemented Egger's linear regression (8) and Begg's rank correlation (9) tests. Additionally, for adjustment of potential funnel plot asymmetry, Fill and Trim analyses were used. Sensitivity analyses were conducted to evaluate the robustness of our findings by excluding studies with a high risk of bias or those that contributed to heterogeneity of the results. All statistical analyses were performed following the methodologies outlined in the *Cochrane Handbook for Systematic Reviews of Interventions* (10).

## Results

### Study selection

A systematic literature search was conducted across databases such as PubMed, EMBASE, and Web of Science, identifying a total of 1,233 records. An additional 42 records were obtained through other sources, bringing the total number of identified records to 1,275. After removing 321 duplicates, we screened 954 unique records based on their titles and abstracts, excluding 411 that did not meet the inclusion criteria. This resulted in 543 full-text articles being assessed for their eligibility.

**Figure 1:**
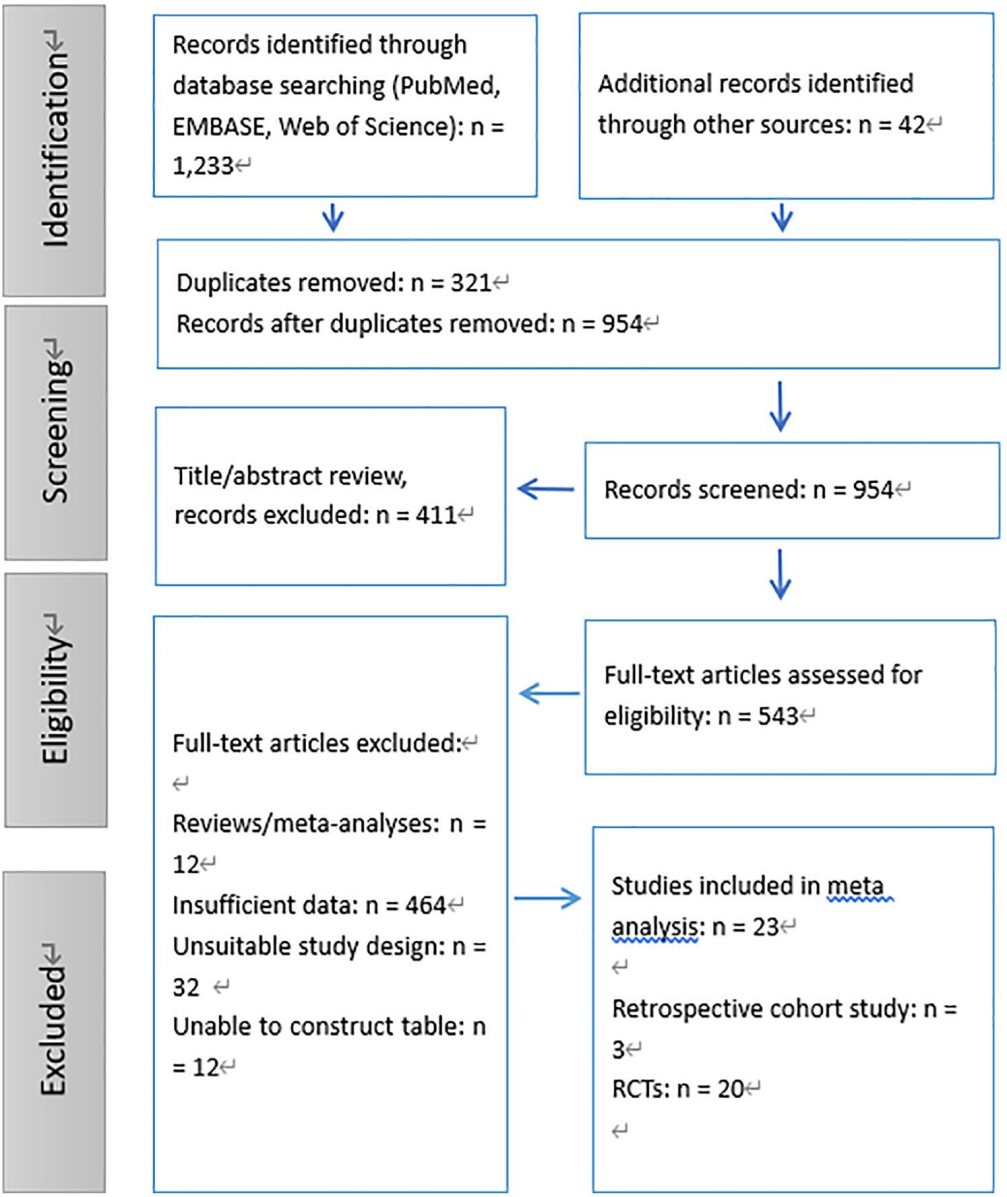
Flow diagram of the study selection process.

Following a thorough review, 520 full-text articles were excluded for various reasons: 12 were reviews or meta-analyses, 464 contained insufficient data, 32 had unsuitable study designs, and 12 could not be used to construct the required tables. Ultimately, 23 studies which followed the inclusion criteria, including 3 retrospective cohort studies (11–13) and 20 RCTs (14–33), were enrolled in the meta-analysis (Figure 1).

### Summary of bias risk

This meta-analysis assessed the risk of bias in studies investigating treatments for GDM using SYRCLE's risk-of-bias tool. The findings indicated that most studies (15 out of 23) had a “low” risk of bias in random sequence generation and allocation concealment, suggesting adequate randomization and fair treatment assignment methods. However, 7 studies were rated as “high” risk for both of these domains, while two2 studies were classified as “unclear.”

Blinding was a large concern, with only 4 studies achieving a “low” risk rating for both blinding of participants/personnel and outcome assessment, whereas “high” risk was rated for 19 studies, demonstrating potential performance and detection bias. Considering incomplete data of outcomes, only 2 studies were rated as “low” risk, 13 were classified as “unclear,” and 8 were deemed “high” risk, raising concerns about data completeness. Selective reporting was generally well managed, with 16 studies rated as “low” risk, though 1 study was classified as “high” risk and 6 as “unclear.”

**Figure 2:**
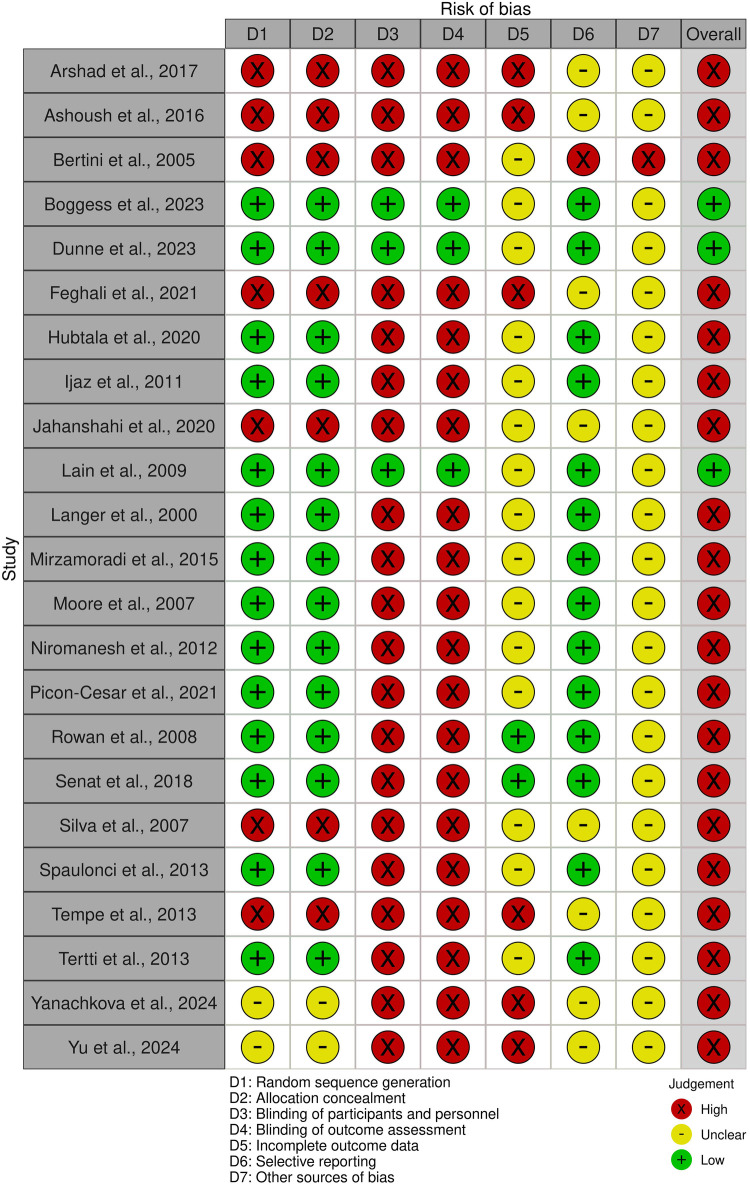
Risk of bias assessment for GDM treatment studies.

Other sources of bias were predominantly marked as “unclear” across 20 studies, with only 3 receiving a “low” risk rating. Overall, a “high” overall risk of bias was rated for 14 studies, “low” for 5 and “unclear” for 4. While some studies demonstrated methodological rigor, these results underscore the need for improved blinding procedures and better handling of incomplete data in future research to enhance the reliability of findings on GDM treatment (Figure 2).

### Comparison of infant birth weight in gestational diabetes treated with oral medications vs. insulin

This meta-analysis evaluated 19 studies comprising a total of 4,664 participants using both fixed-effects and random-effects models. The experimental group (oral medications) was compared as a whole against the control group (insulin). The specific study parameters included the number of studies (*k* = 19) and total number of observations (*o* = 4,664).

**Figure 3:**
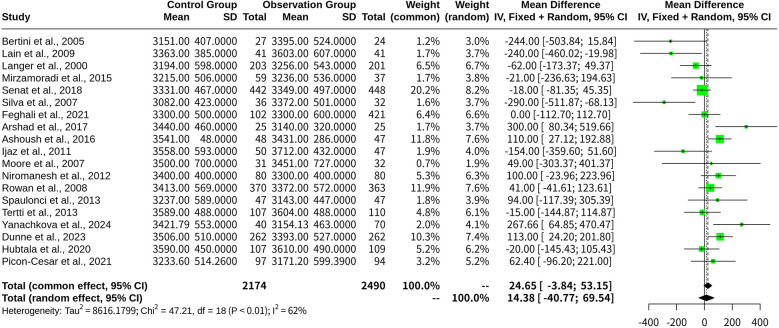
Forest plot for meta-analysis comparing infant birth weight in gestational diabetes treated with oral medications (experimental group) vs. insulin (control group).

In the common-effect model, the MD was 24.6511, with a 95% CI of [−3.8441; 53.1464], a *z*-score of 1.70, and a *p*-value of 0.0900. The random-effects model indicated an MD of 14.3838, 95% CI [−40.7746, 69.5421], *z* = 0.51, and a *p*-value of 0.6093 (Figure 3).

Heterogeneity was quantified as follows: *tau^2^* = 8,616.1799 [3,343.6204; 42,040.9340], *tau* = 92.8234 [57.8240; 205.0389], *I^2^* = 61.9% [37.3%; 76.8%], and *H* = 1.62 [1.26; 2.08]. The results showed a *Q*-statistic of 47.21 (*p*-value, 0.0002; freedom, 18 degrees), indicating heterogeneity across the included studies.

The meta-analytical method employed the inverse variance method, utilizing a restricted maximum-likelihood estimator for *tau^2^* and the Q-profile method to calculate CIs for *tau^2^* and *tau*.

The Trim and Fill analysis added two studies to adjust for funnel plot asymmetry, modifying the random-effects model to an MD of 34.7215 [95% CI: (−28.2624; 97.7054)], with a *z*-score of 1.08 and a *p*-value of 0.2799. This adjustment also recalibrated the heterogeneity measures, yielding *tau^2^* = 14,193.6788, *tau* = 119.1372, *I^2^* = 66.7%, and *H* = 1.73. The *Q*-statistic remained highly significant, with a *p*-value < 0.0001, indicating substantial heterogeneity.

The Egger's regression test for funnel plot asymmetry resulted in a *t*-score of −0.74 with 17 degrees of freedom and a *p*-value of 0.4669, suggesting no evidence of publication bias. Sample estimates showed a bias of −0.6610 with a standard error of 0.8882, an intercept of 62.5957, and an intercept standard error of 56.2853. Additionally, Begg's rank correlation test yielded a *z*-score of −0.91, a *p*-value of 0.3630, and a *ks* estimate of −26.0000 with a standard error of 28.5832, further confirming the absence of publication bias.

### Subgroup analysis on infant birth weight with glyburide and metformin

A random-effects model was used to analyze two subgroups: glyburide (*n* = 7 studies) and metformin (*n* = 12 studies).

For the Glyburide subgroup, the MD was −83.32 [95% CI: (−160.74 to −5.91)], indicating a increase in infant birth weight compared with the overall population. The between-study variance (*tau^2^*) for this subgroup was 4,746.79, corresponding to an inter-study standard deviation of 68.90.

**Figure 4:**
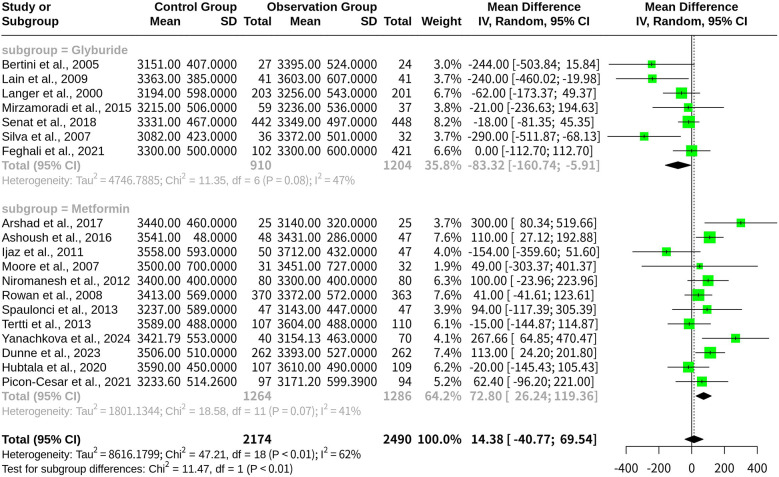
Forest plot for subgroup meta-analysis comparing glyburide and metformin on infant birth weight in gestational diabetes.

For the Metformin subgroup, the MD was 72.80 [95% CI: (26.24–119.36)], suggesting an reduction in infant birth weight relative to the overall population. The between-study variance (*tau^2^*) for this subgroup was 1,801.13, with an inter-study standard deviation of 42.44 (Figure 4).

Heterogeneity statistics for each subgroup indicated moderate variability in the results, with *I^2^* values of 47.1% for glyburide and 40.8% for metformin. These findings highlight notable differences in the impact of Glyburide and Metformin on infant birth weight, emphasizing the importance of treatment selection in GDM management.

### Macrosomia risk comparison: oral medications vs. insulin in gestational diabetes

This meta-analysis included 18 studies with a total of 19,265 observations and 813 events, comparing the probability of macrosomia between the oral medications (experimental group) and the insulin (control group). The common-effect model yielded an OR of 0.7857 with a 95% CI of [0.6815; 0.9059], a *z*-score of −3.32, and a *p*-value of 0.0009, suggesting that oral hypoglycemic medications were associated with a lower risk of macrosomia than insulin. However, when heterogeneity was accounted for, the random-effects model produced an OR of 0.8534 with a 95% CI of [0.6271; 1.1614], a *z*-score of −1.01, and a *p*-value of 0.3134, indicating no statistical difference in the risk of macrosomia between oral hypoglycemic agents and insulin (Table 1).

**Table 1:** Key outcomes from subgroup analysis: glyburide vs. metformin.

Outcome	Medication	OR (95% CI)	*p*-value	Mean difference (g) (95% CI)
Macrosomia	Glyburide	1.3806 (1.0111; 1.8851)	0.04	N/A
	Metformin	0.6728 (0.5728; 0.7903)	<0.0001	N/A
Infant birth weight	Glyburide	N/A	N/A	−83.32 (−160.74; −5.91)
	Metformin	N/A	N/A	72.80 (26.24; 119.36)

**Figure 5:**
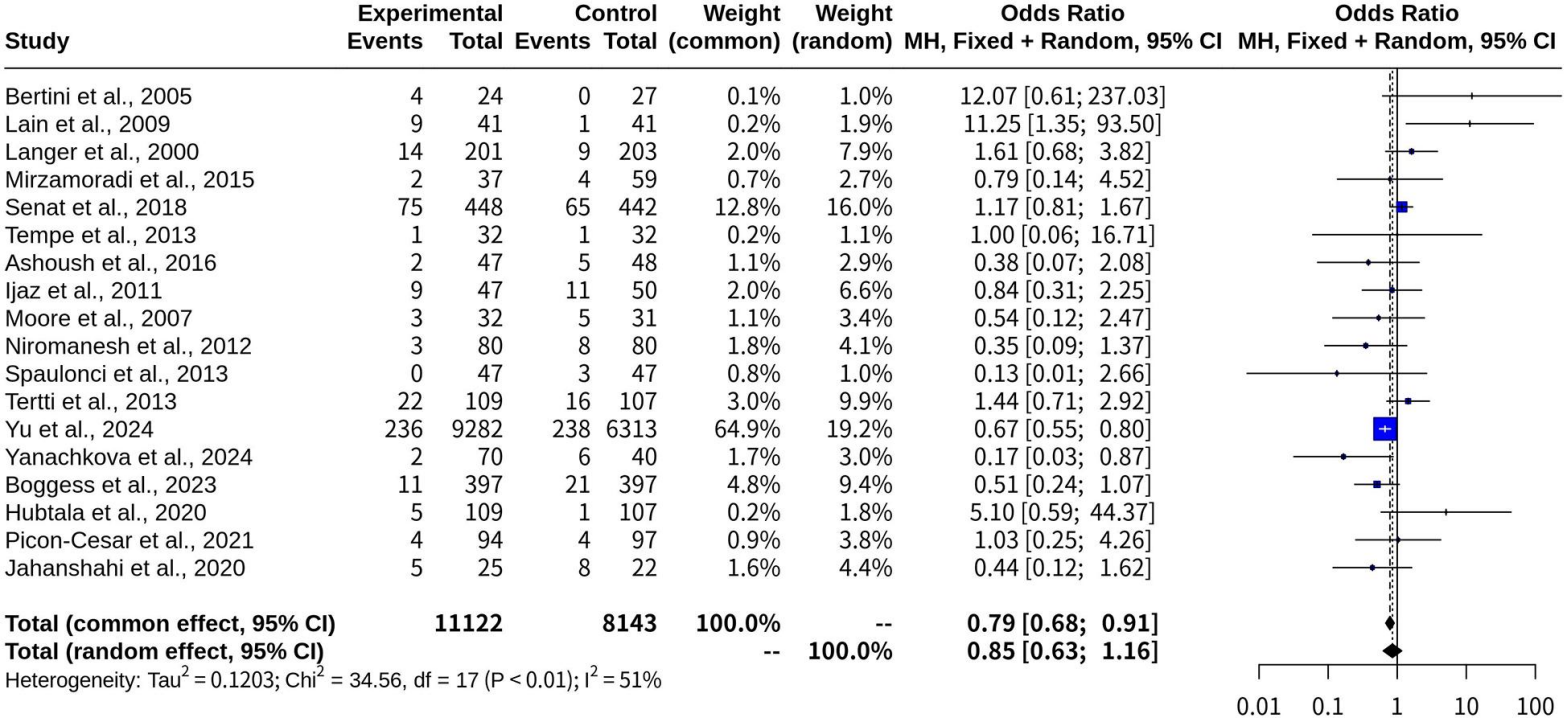
Forest plot for meta-analysis comparing risk of macrosomia with oral medications versus insulin in gestational diabetes.

Heterogeneity was assessed using multiple metrics, yielding *τ*^2^ = 0.1203 [0.0147; 2.1179], *τ* = 0.3469 [0.1213; 1.4553], I^2^ = 50.8% [15.5%; 71.4%], and H = 1.43 [1.09; 1.87], suggesting moderate heterogeneity across the included studies. The Q-statistic was 34.56 with 17 degrees of freedom and a *p*-value of 0.0071, confirming the presence of heterogeneity (Figure 5).

These findings indicate that while the common-effect model suggests a potential benefit of oral medications in reducing macrosomia risk, the variability among studies weakens this conclusion when analyzed using the random-effects model. Further high-quality research may be needed to clarify this relationship and determine whether specific oral agents contribute differently to macrosomia risk.

### Subgroup analysis on macrosomia risk with glyburide and metformin

**Figure 6:**
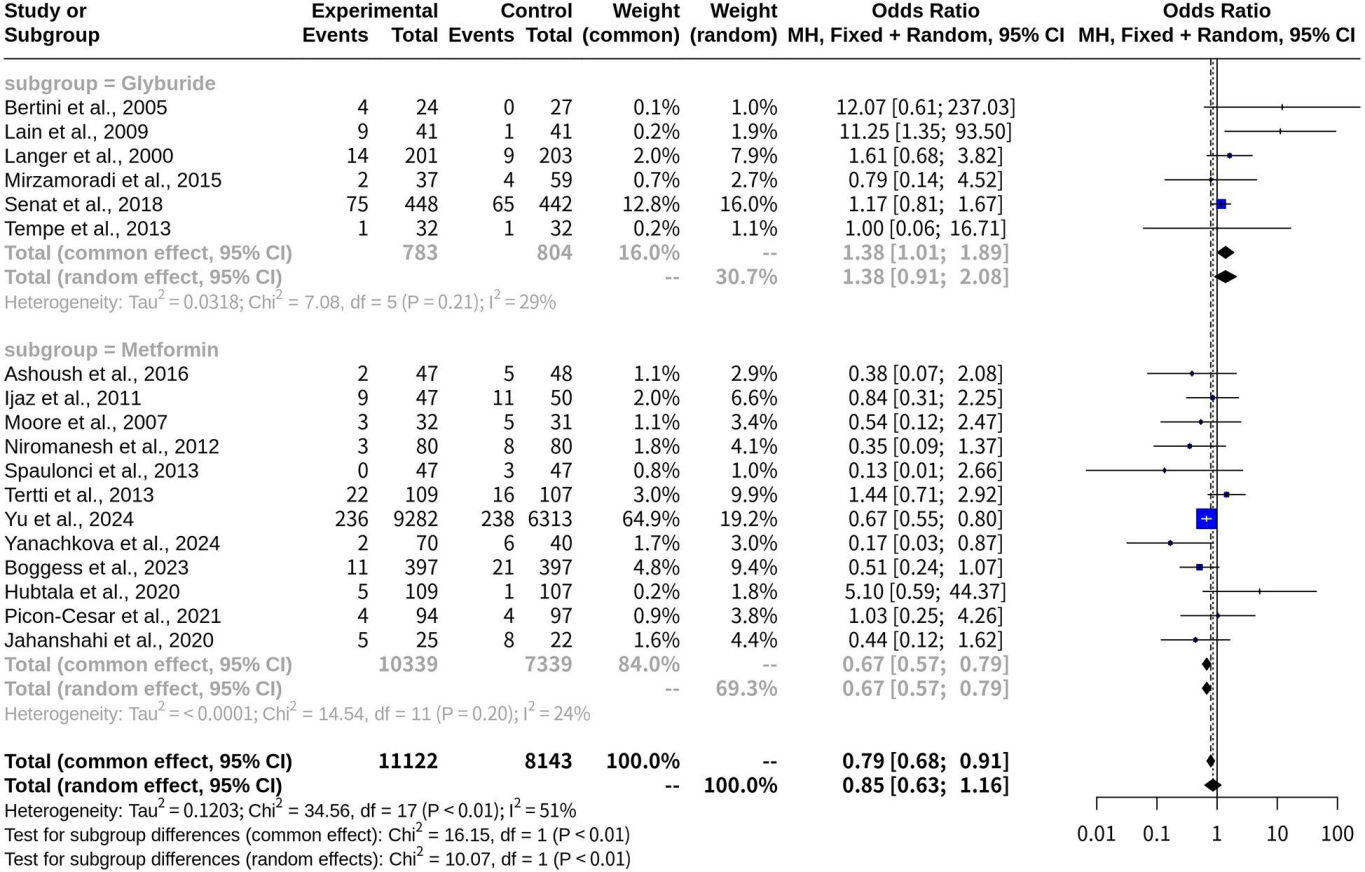
Forest plot for subgroup meta-analysis comparing glyburide and metformin on risk of macrosomia with oral medications versus insulin in gestational diabetes.

The subgroup analysis compared the ORs and 95% CIs for macrosomia risk between the Glyburide and Metformin treatment groups. In the common-effect model, the risk of macrosomia in patients receiving Glyburide was increased compared to those not on Glyburide (OR, 1.3806; 95% CI: 1.0111; 1.8851). The heterogeneity measure, *I^2^*, was 29.4%, suggesting moderate heterogeneity among the studies in this subgroup. Conversely, patients taking metformin had an OR of 0.6728 [95% CI: (0.5728; 0.7903)], demonstrating a reduced risk of macrosomia compared with those not on metformin. The heterogeneity measure for this subgroup was lower, with *I^2^* at 24.3% (Figure 6). A test for subgroup differences confirmed that the difference between the Glyburide and Metformin subgroups was statistically significant (*p*-value < 0.0001). When the random-effects model was applied, similar trends were observed, though slight variations in ORs and CIs were noted due to the incorporation of heterogeneity into the analysis (Table 1).

## Discussion

In this meta-analysis, we reviewed 23 studies to compare the efficacy of oral medications vs. insulin in managing gestational diabetes mellitus (GDM), focusing on the risk of macrosomia and infant birth weight. Our findings indicate that, overall, there was no statistical difference in the risk of macrosomia between oral medication and insulin treatment.

However, subgroup analyses revealed the distinct effects of individual medications. Glyburide was associated with an increased risk of macrosomia (OR = 1.3806, *p* < 0.05), whereas metformin was linked to a reduced risk (OR = 0.6728, *p* < 0.0001). Similarly, although no statistical overall difference was found in infant birth weight between oral medications and insulin, subgroup analysis showed that glyburide was associated with lower birth weights, whereas metformin was linked to higher birth weights within their respective subgroups. These findings emphasize the need for personalized treatment approaches in GDM management, as different oral medications may have contrasting effects on fetal growth and neonatal outcomes. The underlying mechanisms of these differences and their long-term clinical implications for both mothers and their offspring remain to be further investigated.

Glyburide, a sulfonylurea, which can stimulate pancreatic β-cells to secret insulin, is primarily used to decrease blood glucose levels. While effective in managing maternal blood sugar, this mechanism can lead to overproduction of insulin, potentially promoting fetal growth and thereby increasing the risk of macrosomia (16). This may also explain the highe birth weights in infants exposed to glyburide, possibly due to its impact on the intrauterine environment or the level of glycemic control during pregnancy (14).

In contrast, metformin primarily improves insulin sensitivity and reduces hepatic glucose output. Unlike glyburide, metformin's mechanism may reduce excessive fetal growth stimulation by lowering maternal insulin resistance, which could decrease the incidence of macrosomia (24). By potentially reducing fetal exposure to excess nutrients and growth hormones, metformin may contribute to higher birth weights due to a healthier intrauterine environment rather than pathological growth (23).

Although metformin can cross the placenta, its impact on fetal development appears to be less direct than that of glyburide. Glyburide may directly influence fetal insulin levels and growth, potentially explaining the lower macrosomia risk observed in the metformin group (17).

However, variability in patient selection, adherence to treatment protocols, and measurement methods across studies could affect these findings. For instance, women treated with glyburide might have had higher baseline blood sugar levels or additional comorbidities at the start of the study, which could have influenced maternal and neonatal outcomes (18). A significant limitation was the lack of blinding in most studies (19 out of 23). This absence of blinding of participants, personnel, and outcome assessors introduces the potential for performance and detection bias. For instance, knowledge of the treatment arm could unconsciously influence the behavior of participants (e.g., adherence to diet) or healthcare providers (e.g., more frequent glucose monitoring), thereby affecting the reported outcomes. This lack of blinding could also lead to subjective differences in how outcomes like birth weight are measured or recorded, potentially skewing the effect estimates. These factors highlight the complexity of comparing treatment effects and underscore the importance of individualized management strategies for patients with GDM.

The observed moderate heterogeneity, as indicated by the I^2^ statistics, likely stems from multiple unaddressed clinical and methodological differences among the included studies. First, diagnostic criteria for GDM vary globally (e.g., one-step vs. two-step screening protocols), leading to different patient populations. Second, maternal characteristics such as baseline BMI, which is a significant risk factor for macrosomia, were not consistently reported, nor were the individualized treatment targets for glycemic control. Furthermore, a mix of study designs (19 RCTs and 3 retrospective cohort studies) and differences in geographical locations may also contribute to the observed variability. Although our subgroup analyses by medication type addressed a key source of heterogeneity, a more comprehensive meta-regression or stratified subgroup analysis based on these clinical and methodological factors would be necessary to fully explore their influence on the outcomes. However, the data available in the included studies did not allow for such detailed analyses.

Differences in the effectiveness and consistency of blood glucose control between glyburide and metformin may contribute to their distinct effects on pregnancy outcome. By providing more stable glucose control, metformin may beneficially limit fetal growth, leading to lower birth weights. Conversely, glyburide may be associated with greater glucose fluctuations that could promote excessive fetal growth (25). These differences likely reflect fundamental variations in the physiological effects, pharmacokinetics, and maternal-fetal impacts of these two drugs. While no significant overall difference was found compared with insulin, subgroup analyses suggested that glyburide and metformin led to different pregnancy outcomes (28). Further research is necessary to better understand these dynamics, potentially guiding the development of more personalized treatment approaches for GDM tailored to individual patient characteristics and drug pharmacologic profiles (29).

Based on our meta-analysis, the differential effects of glyburide and metformin on macrosomia and infant birth weight have important clinical implications. Given its association with a reduced risk of macrosomia and lower birth weights, metformin may be a preferred first-line therapy, particularly in pregnant individuals at a higher risk of fetal macrosomia. This aligns with recent guidelines from the American Diabetes Association and the National Institute for Health and Care Excellence, which recommend metformin as a first-line agent for GDM where feasible. However, glyburide may still be considered an appropriate alternative for patients who cannot tolerate metformin due to gastrointestinal side effects or for whom metformin is contraindicated. These findings underscore the need to move beyond a one-size-fits-all approach to GDM treatment, favoring a personalized strategy that accounts for individual patient risk factors and the distinct pharmacological profiles of these oral medications.

## Limitations

There are also several limitations. First, heterogeneity was notable, as reflected by the I^2^ statistics, indicating variability in study designs, patient populations, and outcome definitions, which may limit the generalizability of our findings. Second, the number of studies in each subgroup (Glyburide and Metformin) was limited, potentially reducing the statistical power to detect subtle differences or confirm the consistency of subgroup findings. Additionally, reliance on published data introduces the possibility of publication bias, despite efforts to mitigate this through funnel plot analyses and statistical tests. Furthermore, many of the included studies lacked long-term follow-up data, restricting our ability to assess the long-term effects of these medications on maternal and child health outcomes. Finally, the retrospective nature of some studies may introduce recall or selection bias, potentially skewing the results.

These limitations highlight the need for future well-designed, large-scale randomized controlled trials with longer follow-up to better assess the safety and efficacy of oral medications in GDM management.

## Conclusion

This meta-analysis of 23 studies evaluating the use of oral hypoglycemic agents in GDM suggests that while no significant overall difference was found in the risk of macrosomia or infant birth weight compared to insulin, subgroup analyses indicated differential impacts depending on the type of oral medication used. Glyburide appears to increase the risk of macrosomia and is associated with higher birth weights, whereas metformin seems to reduce the macrosomia risk while correlating with lower birth weights. These findings underscore the complexity of GDM management and emphasize the importance of considering the pharmacological properties of medications when selecting treatment options.

## Data Availability

The original contributions presented in the study are included in the article/Supplementary Material, further inquiries can be directed to the corresponding author.
